# COVID-19 Vaccine Knowledge, Attitude, Acceptance and Hesitancy among Pregnancy and Breastfeeding: Systematic Review of Hospital-Based Studies

**DOI:** 10.3390/vaccines11111697

**Published:** 2023-11-07

**Authors:** Vincenza Gianfredi, Alessandro Berti, Pasquale Stefanizzi, Marilena D’Amico, Viola De Lorenzo, Lorenza Moscara, Antonio Di Lorenzo, Vincenzo Venerito, Silvana Castaldi

**Affiliations:** 1 Department of Biomedical Sciences for Health, University of Milan, Via Pascal, 36, 20133 Milan, Italy; vincenza.gianfredi@unimi.it (V.G.); alessandro.berti@unimi.it (A.B.); marilena.damico@unimi.it (M.D.); viola.delorenzo@unimi.it (V.D.L.); silvana.castaldi@unimi.it (S.C.); 2 Interdisciplinary Department of Medicine, University of Bari Aldo Moro, Piazza G. Cesare 11, 70121 Bari, Italy; lorenza.moscara@gmail.com (L.M.); antoniodilorenzo95@gmail.com (A.D.L.); 3 Rheumatology Unit, Department of Precision and Regenerative Medicine, Jonic Area, University of Bari “Aldo Moro”, 70121 Bari, Italy; vincenzo.venerito@uniba.it; 4 Fondazione IRCCS Ca’ Granda Ospedale Maggiore Policlinico, Via Francesco Sforza, 35, 20122 Milan, Italy

**Keywords:** vaccine hesitancy, pregnancy, adverse events, communication, SARS-CoV-2, COVID-19

## Abstract

The risk of unfavourable outcomes for SARS-CoV-2 infection is significant during pregnancy and breastfeeding. Vaccination is a safe and effective measure to lower this risk. This study aims at reviewing the literature concerning the anti-SARS-CoV-2 vaccine’s acceptance/hesitancy among pregnant and breastfeeding women attending hospital facilities. A systematic review of literature was carried out. Hospital-based observational studies related to vaccination acceptance, hesitancy, knowledge and attitude among pregnant and breastfeeding women were included. Determinants of acceptance and hesitancy were investigated in detail. Quality assessment was done via the Johann Briggs Institute quality assessment tools. After literature search, 43 studies were included, 30 of which only focused on pregnant women (total sample 25,862 subjects). Sample size ranged from 109 to 7017 people. Acceptance of the SARS-CoV-2 vaccine ranged from 16% to 78.52%; vaccine hesitancy ranged between 91.4% and 24.5%. Fear of adverse events for either the woman, the child, or both, was the main driver for hesitancy. Other determinants of hesitancy included religious concerns, socioeconomic factors, inadequate information regarding the vaccine and lack of trust towards institutions. SARS-CoV-2 vaccine hesitancy in hospitalized pregnant women appears to be significant, and efforts for a more effective communication to these subjects are required.

## 1. Introduction

Pregnant and breastfeeding women encounter distinct challenges concerning vaccine acceptance [1]. Throughout this crucial period, women proactively seek information concerning their health and their child’s well-being, with significantly impacts their medical decisions [2]. Vaccination holds particular importance for this population group, as certain vaccine-preventable diseases can lead to severe outcomes during pregnancy or pose risks to the child both before and after birth [3]. Various interconnected factors influence vaccine acceptance encompassing individual’s knowledge and attitudes towards vaccination, societal norms, and perceptions of benefits and risks related to vaccination [4]. Vaccine hesitancy, characterized by delays in completing vaccination schedules or in refusing vaccines, can emerge when individuals lack sufficient motivation to get vaccinated [5]. Numerous factors contribute to this reluctance, including concerns about safety and effectiveness, as along with mistrust in vaccine development and regulatory processes [6,7].

The emergence of the COVID-19 pandemic brought about additional uncertainties surrounding health choices and vaccination [8]. During the initial months of the pandemic, there was a lack of both effective therapy and a safe and effective vaccine. This created substantial expectations within the general population for a vaccine capable to “restore normalcy” [9]. Concurrently, as anti-SARS-CoV-2 vaccines became available, the distinctive characteristics of these vaccines, coupled with the limited initial data regarding their long-term safety and efficacy, along with the remarkably rapid pace of their development, engendered feelings of uncertainty and ambivalence toward vaccination. Pregnant and breastfeeding women, in particular, expressed concerned about possible side effects of the vaccine affecting either them or their child [10]. Despite these concerns, subsequent data from the Centers for Disease Control and Prevention reassured the safety of COVID-19 vaccines for pregnant women, promoting public health programs to prioritize their vaccination [3,11]. It is essential to recognize that vaccine hesitancy often varies depending on the specific vaccine and the socio-cultural background of the hesitant individual. Additionally, COVID-19 vaccines are relatively new, making it challenging to predict the evolution of hesitancy towards them. To gain a deeper understanding of the factors contributing to COVID-19 vaccine hesitancy among pregnant and breastfeeding women, we planned a systematic review.

The primary aim of this review is to synthesize existing literature on knowledge, beliefs, attitudes, barriers, and facilitators related to COVID-19 vaccine acceptance among pregnant and breastfeeding women. The review specifically seeks to address two key research questions: (1) What is the level of knowledge regarding COVID-19 vaccination among pregnant/breastfeeding women? (2) What are the facilitators and barriers to COVID-19 vaccine acceptance associated with pregnancy and/or breastfeeding? The findings from this review will provide valuable insights into the current landscape and help identify research gaps, informing public health strategies to promote vaccination in this population. For consistency, this review focuses on hospital-based studies, while population-based studies have been addressed separately, considering potential differences in health literacy, attitudes towards healthcare, and trust in medical professionals among women seeking medical support.

## 2. Materials and Methods

The systematic review adhered to the guidelines established by the Cochrane Collaboration [12] and followed the Preferred Reporting Items for Systematic Reviews and Meta-Analyses (PRISMA) 2020 guidelines [13] for transparent reporting.

### 2.1. Search Strategy and Data Collection

To construct a comprehensive search strategy, we logically linked a combination of free text words and Medical Subject Headings (MeSH) terms using Boolean operators. This approach was implemented for each database, with simultaneous searches by two independent authors. In brief, keywords referred to breastfeeding/pregnant women (and synonyms) were combined with keywords related to knowledge, attitude, acceptance and hesitancy (and synonyms). A detailed search strategy has been previously published [14]. Additionally, we screened the reference lists of included articles to identify potentially relevant studies that may have been missed.

### 2.2. Inclusion and Exclusion Criteria

Eligible studies met the following criteria: (i) original observational hospital-based studies (cross-sectional, case-control, or cohort studies), (ii) conducted after 2019, (iii) focusing on knowledge, attitudes, acceptance and hesitancy related to COVID-19 vaccination, (iv) involving pregnant or breastfeeding women, (v) published in English peer-reviewed international journals. The exclusion criteria encompassed: studies not conducted on humans or those involving a different population, studies combining data with different and multiple outcomes or assessing outcomes not listed in our inclusion criteria (e.g., vaccine efficacy/safety/development or collecting serological/immunological data); articles assessing acceptance/hesitancy/refusal against vaccines other than COVID-19; articles not written in English or not published in peer-reviewed international journals; non-observational studies, e.g., trials (randomized or non-randomized controlled trials); and, lastly, non-original research papers, including reviews or meta-analyses, articles lacking quantitative information or details, and non-full-text papers (e.g., letters to the editor, conference papers, commentary notes, expert opinions, abstracts). The selected inclusion/exclusion criteria were established based on our research question. Specifically, original observational hospital-based studies were included because they provide valuable data on the knowledge, attitudes, acceptance, and hesitancy related to COVID-19 vaccination in pregnant or breastfeeding women focusing on the hospital setting. Secondly, we applied a time lag, selecting article published after 2019, given that the COVID-19 pandemic began at the end of 2019. This ensures that the data is current and relevant to the ongoing situation. Thirdly, we focused on knowledge, attitudes, acceptance and hesitancy to specify the topic of interest. Fourthly, we defined our population of interest as pregnant or breastfeeding women, ensuring that the selected studies include this specific population Additionally, we limited our selection to articles published in English and in peer-reviewed international journals because English is the internationally recognized language for scientific publication, and we have confidence that high-quality articles are typically published in peer-reviewed international journals. Peer-reviewed international journals that, in turn, usually offer a quality assurance measure.

### 2.3. Selection Process

The complete set of retrieved studies was imported into EndNote software (EndNote^®^ for Microsoft, X9 version, Redmond, WA, USA, 2020), and duplicates were initially removed using automated tools, followed by manual cross-checking. The remaining articles underwent a two-step evaluation: first based on title and abstract, followed by full-text assessment.

### 2.4. Data Extraction

Data extraction was carried out by two reviewers using a predefined Excel spreadsheet (Microsoft Excel^®^ for Microsoft 365 MSO, 17 version, Redmond, WA, USA, 2019). The extracted information included author details, study characteristics, study population, assessment tools, recruitment methods, outcomes, methodological details, and statistical analyses. Additional details about data extraction are reported into the protocol [14]. If studies report data using risk estimates, for instance, odds ratio (OR), risk ratio (RR) or hazard ratio (HR), the maximally adjusted data, along with the list of variables used for the adjustment were recorded. Lastly, information on received research funding and conflict of interests was also extracted.

### 2.5. Quality Assessment

Two independent reviewers assessed the risk of bias using the Joanna Briggs Institute (JBI) quality assessment tools [15] which consists of eight items scored on a scale from −2 to 2. Based on the cumulative scores, studies were categorized as low (score from −16 to 4), moderate (score from 5 to 9), or high quality (score more than 10 and up to 16), based on the JBI instruction [15].

## 3. Results

### 3.1. Literature Search

**Figure 1:**
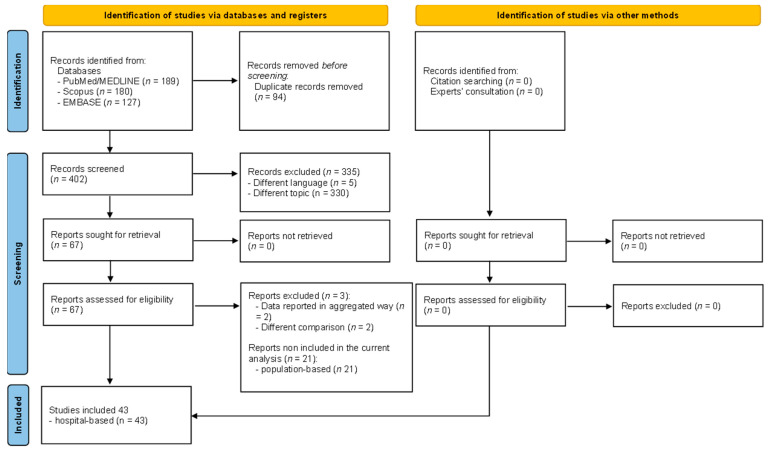
PRISMA flow diagram detailing the selection process.

Initially, a total of 496 records were retrieved searching on PubMed/Medline, Scopus and EMBASE. Consultation with experts did not add any further eligible studies. After performing duplicate screening using Endnotes, 94 records were removed. Subsequently, based on language and on title/abstract screening, a total of 67 articles were considered eligible. However, after full-text assessment, three articles were removed due to aggregated data [16] and different comparison [17,18]. Because of the high heterogeneity of the remaining 64 records, we chose to present results separately for population-based studies (21 records included; previously published [19]) and results of hospital-based studies (43 records included [2,20,21,22,23,24,25,26,27,28,29,30,31,32,33,34,35,36,37,38,39,40,41,42,43,44,45,46,47,48,49,50,51,52,53,54,55,56,57,58,59,60,61]; reported in the current manuscript). The disagreement about reviewers during the selection process was around 10%. All the disagreements were solved through discussion among the two. The final full screening process is detailed in Figure 1.

### 3.2. Main Characteristics of Included Studies

Approximately two third of the included studies (*n* = 27/43) were conducted in 2021, approximately one third of the included studies (*n* = 13/43) were conducted in 2022, and three studies were conducted in 2020. The vast majority (*n* = 37/43) were cross-sectional studies, while the remaining six were cohort studies (*n* = 6/43). All the studies were performed in gynecological or maternal units at public or private hospitals. Recruitment was performed by inviting consecutive women attending the clinic in approximately half of the cases (*n* = 25/43) [2,20,24,25,26,29,30,33,35,37,38,39,40,42,44,46,47,49,51,52,53,54,56,60,61], random sampling was employed in four studies (*n* = 4), antenatal care registry was used in three studies (*n* = 3) [21,36,59], convenience sampling was adopted in other three studies (*n* = 3) [27,28,32], multistage sampling approach was used in two studies (*n* = 2) [55,58], the snow-ball method was used in one study (*n* = 1) [22], and one study used data from an ongoing prospective longitudinal cohort study (*n* = 1) [50]. Four studies did not specify the recruitment method adopted [23,31,45,48].

In approximately half of the studies, questionnaires were administered face-to-face (*n* = 21/43) [2,21,26,30,34,35,38,39,40,41,42,43,44,45,46,47,48,56,58,61], however, two of them combined the face-to-face interview with the on-line administration [44,61]. Seven studies used on-line administration [24,27,28,29,33,54,60], five studies performed a self-administration (paper-based) [22,32,37,49,51] and two studies used a telephone administration [36,50]. Eight studies did not report information regarding administration methods [20,23,25,31,52,53,55,59].

Regarding tools used to assess the outcome of interest, in the vast majority of the included studies, authors developed ad hoc questionnaire (*n* = 37/43). Eectronic medical records were used in two studies [23,59], while two studies used a pre-developed questionnaire (respectively the Vaccination Attitude Examination scale [28] and the Attitude toward COVID-19 vaccine scale [39]). Lastly, the remaining two studies did not provide the information [35,42]. Regarding validation of the tools adopted, approximately half of the sample (*n* = 21/43) used validated tools, while five studies did not report the information [2,23,36,46,54].

Regarding source of funds, approximately one third of the included studies (n = 19/43) did not receive funds [21,23,27,28,29,30,31,34,36,37,38,39,42,43,44,45,53,56,57]. Eight studies declared source of funds [32,33,49,50,51,54,55,61], whereas, the remaining did not report the information. However, almost all the included studies (*n* = 38/43) declared no conflict of interest, while the remaining did not report the information [24,37,46,47,52]. Details are provided in Table 1.

### 3.3. Main Characteristics of Studied Population

The vast majority of included studies (*n* = 30/43) exclusively recruited pregnant women, while four studies focused solely on postpartum/breastfeeding women [25,41,48,50]. The remaining studies included both pregnant and postpartum/breastfeeding women [20,22,24,39,40,51,52,57,61]. Women’s ages were reported as mean and standard deviation, or mean and interquartile range, or range, or percentage; however, the youngest women were 18 years old, while the oldest were 49 years old. The smallest sample size was 109 [53], whereas the largest was 7017 [59], and attrition rate ranged between 0% and 43%. Details can be found in Table 2.

### 3.4. Knowledge and Attitude toward COVID-19 Vaccine

Knowledge was assessed in 6 studies [21,26,36,42,43,57], and good level of knowledge ranged between 18.88% [57] to 88.2% of the populations under examination [21]. However, it is important to note that none of the retrieved studies specifically focused solely on knowledge. Instead, knowledge assessment was part of a broader assessment, often combined with reasons for accepting or refusing the COVID-19 vaccine. Therefore, no information regarding potential predictor of level of knowledge has been retrieved.

Attitude toward COVID-19 vaccine was explored in 8 studies [21,25,26,39,41,42,43,57]. Among these, 5 studies reported positive attitude expressed as overall percentage of the populations being studied (ranging from 38.54% to 68.2%) [21,26,42,43,57]. One study, reported percentage of attitude separated for each assessed aspect (level of immunity 60.8%, number of vaccination 60.3% and type of vaccine 53.3%) [25]. One study expressed attitude as a mean score [41], and lastly one did not report the value [39]. Out of 8 studies, only two of them explored potential predictors of attitude [39,41]. Specifically, fear of getting the infection [41], pregnancy at risk [41], and consulting not official sources of data (sources of data different from, as for instance, Governmental or Health Agency/Authority) [41] were all associated with higher positive attitude. Not knowing the recommendation [41], lower level of education, and no history of COVID-19 infection were associated with a lower positive attitude [41], while living in urban area was associated with lower positive attitude in another study [39]. Belief that COVID-19 vaccine is safe and postponing vaccination after delivery were all associated with lower rates of people reporting negative attitude [39]. The need to receive information on COVID-19 vaccine [41], marital status [41], health status [41], and planned pregnancy [39] were not found to be associated with attitude.

### 3.5. COVID-19 Vaccine Acceptance

A total of 33 articles estimated the COVID-19 vaccination acceptance among pregnant/breast-feeding women, with rates ranging from 16% [56] to 78.52% [40]. Out of these 33 articles, 27 studies further explored the association/correlation/differences between vaccine acceptance and several predictors, as detailed in Table 3. Specifically, we identified socio-demographic data, lifestyle factors, health-related aspects, pregnancy characteristics and COVID-19 related aspects as topics studied in association with COVID-19 vaccine hesitancy among pregnant/breastfeeding women.

#### 3.5.1. Socio-Demographic Data

Among socio-demographic factors, the following were retrieved: maternal age (n = 10), educational level (n = 13), ethnicity (n = 3), employment (n = 8; work-related stress n = 1); area of residency (n = 5), income (n = 5), marital status (and husband’s characteristics n = 4), and cohabitation (n = 5) were retrieved.

Regarding maternal age, half of the retrieved studies did not find any significant association, whereas the remaining 5 studies found that age ≥ 35 years was significantly associated with higher COVID-19 vaccine acceptance. Out of a total of 13 studies, 8 reported a statistically significant association: higher education was linked to higher acceptance, while lower education was linked to lower acceptance (the association was not significant in the remaining 5 studies). Considering ethnicity, all three studies identified a significant association between being a member of a minority group and higher COVID-19 vaccine acceptance. Most included studies (6 out of 8) did not detect an association between acceptance and employment, while the remaining two found a positive association between being employed and COVID-19 vaccine acceptance. Moreover, feeling overloaded [54] but not work-related stress [54] was associated with higher acceptance rate. Living in urban area was associated with higher acceptance, except in one study that did not find a significant association between living in rural area and vaccine acceptance [34]. Income was not associated with acceptance, except in one study that found a significant association between lower income and lower COVID-19 vaccine acceptance [23]. Marital status was directly assessed in one study that did not find an association with acceptance. However, husband’s educational level [58], living with husband and children [47], and having a husband who favoured COVID-19 vaccination [49] were all significantly associated with a higher rate of acceptance. Moreover, living with or being in contact with people vaccinated against COVID-19 or being in favour of receiving the vaccine was significantly associated with COVID-19 vaccine acceptance in all the included studies, except in one study [21]. Furthermore, living with people older than 65 years or the number of householder members in general were not associated with acceptance [35].

#### 3.5.2. Lifestyle Factors

Alcohol and smoking habits were explored in one and two studies, respectively, and none of them detected a significant association with COVID-19 vaccine acceptance. Considering religion; two studies assessed the association with acceptance, but only one of them found an association between Muslim religion and lower acceptance [40]. Lastly, caring about travelling was associated with higher acceptance [27], while a high level of perceived cues to action was associated with higher acceptance rate [55].

#### 3.5.3. Health Related Aspects

Only one study assessed the association between BMI and acceptance, but no significant association was found [23]. Comorbidities/health status was explored in 10 studies, half of them found a significant association between a history of chronic diseases and higher acceptance, while the remaining studies did not find an association. Having a private health insurance was explored in one study, which found an association with a lower acceptance rate [47]. A history of COVID-19 infection was assessed in 6 studies, but only one found an association with higher acceptance [41]. All the remaining did not find a significant association. Moreover, a high level of perceived susceptibility was assessed in one study and appeared to be significantly associated with higher acceptance rate [55]. Lastly, higher adherence to mitigation measures against COVID-19, having received information/recommendations about COVID-19 vaccine, and having received other vaccinations (such as influenza or pertussis) during pregnancy were significantly associated in all the assessed studies (3, 4 and 3 studies, respectively).

#### 3.5.4. Pregnancy Characteristics

Gestational week was assessed in 8 studies, of which 6 studies found an association between a later gestational week and higher COVID-19 acceptance, whereas two studies failed to find a statistically significant association [35,45]. Parity was assessed in 8 studies but only one study found a statistical association between a higher number of pregnancy and higher acceptance [40]; all the remaining studies did not find a significant association. Pregnancy at risk was only assessed in one study and was not found to be associated with acceptance [41]. History of abortion was explored in two studies, of which one study found an inverse association with acceptance [59], while the second did not find an association [21]. Lastly, a poor obstetric history was associated with lower acceptance rate, insufficient prenatal care was in one study associated with lower acceptance [59] but was not significant in another study [21]; while infertility treatment was associated with higher acceptance [59].

#### 3.5.5. COVID-19 Related Aspects

Regarding COVID-19 related aspects, we detected the following topics: fear of COVID-19, knowledge, attitude toward vaccination, perceived barrier, safety and benefit of vaccination, vaccine efficacy, facility, data availability and source of data, and trust in authorities.

Fear of COVID-19 infection was assessed in 5 studies, of which three found an association with higher vaccine acceptance, while this association was not found in the other two studies [41,61]. A higher level of knowledge on COVID-19 or its vaccine was associated with a higher acceptance rate in 6 studies, except in one that failed to find a statistical association [45], 2022. Attitude toward COVID-19 vaccination was assessed in 6 studies. Positive attitude was associated with higher acceptance in 5 out of 6 studies; only one study did not detect a significant association [43]. Perceived barriers were explored in one study, which found a positive association between lower level of perceived barriers and higher acceptance rate. The perception of vaccine benefits was assessed in three studies, all of which concurred in finding an association between higher benefit perception and higher acceptance rates. COVID-19 vaccine safety for women or fetus was explored in 6 studies. Fear of side effects, vaccine’s toxicity, or belief that vaccine can cause the infection were all associated with lower acceptance, except in one study where fear of side effects was not associated with acceptance [36]. General confidence in vaccine safety was also not associated with acceptance [49]. On the contrary, confidence in vaccine efficacy was assessed in three studies and all of them found a significant association with higher acceptance. Similarly, access to vaccination centres was also associated with acceptance [27]. Regarding data availability and the source of data, studies found that the perceived unavailability of data on COVID-19 vaccines, the feeling that trials were rushed, and the belief that people from minority groups were not adequately represented in trials were all associated with lower acceptance. On the contrary being exposed to official source of data was associated with higher acceptance. Lastly, trust in authorities (government and vaccine features) was associated with higher acceptance.

### 3.6. COVID-19 Vaccine Hesitancy

A total of 23 studies assessed the COVID-19 vaccine hesitancy, which ranged between 91.4% and 24.5%. The main reasons for refusing vaccines included fear of side effects and concerns on vaccine safety (including fear of infertility~20.5% [46], or risk of death ~7.9% [31]) for both women (ranging between 31.4% [39]–73% [56]) or babies (22.5% [39]–91.7% [61]). Concerns on vaccine efficacy/effectiveness were also common reasons (32.4% [48]–58.0% [26]), along with lack of data on vaccine safety/efficacy (6.7% [31]–76.0% [48]). More details are reported in Supplementary Table S1. Predictors of vaccine hesitancy are discussed below and summarized in Table 3.

#### 3.6.1. Socio-Demographic Data

Among socio-demographic data, maternal age (n = 5), educational level (n = 7), ethnicity (n = 3), employment (n = 3); area of residency (n = 1), income (n = 3), marital status (and husband’s characteristics n = 2), and cohabitation (n = 2) were retrieved.

Considering maternal age, three out of five studies did not find any significant association, whereas the remaining two found a significant associated between older age and a lower rate of hesitancy. Educational level was also not statistically significantly associated with hesitancy in most of the retrieved studies (5 out of 7). In the remaining two studies, one found an association between higher level of education and lower level of hesitancy [2], whereas the last one found an inverse association [41]. Ethnicity (minorities) was associated with lower rate of hesitancy in two out of three study, but DesJardin et al. [30] failed to find a significant association. On the contrary, employment was not associated with hesitancy in all the three retrieved studies [30,31,38]. Similarly, area of residency [46] and income [30,31,46] were not associated with hesitancy. Marital status [30] and husband’s level of education [46] were also not significantly associated with hesitancy. Lastly, the number of household members and in particular living with school children, significantly differed among hesitant and not hesitant women [48], as well as being in contact with other pregnant women vaccinated was significantly associated with lower rate of hesitancy. However, living with people affected by comorbidities did not predict COVID-19 vaccine hesitancy [48].

#### 3.6.2. Lifestyle Factors

Drugs consumption [30] and smoking habit [2,31] were explored in one and two studies, respectively. However, only one study found a significant association between smoking habit and higher rate of hesitancy [2], while all the other did not find an association. Lastly, a high level of perceived cues to action, but not self-efficacy [32], was associated with lower hesitancy [32].

#### 3.6.3. Health Related Aspects

Comorbidities/health status [2,32] and a history of COVID-19 infection [30,50] were each examined in two studies, and no association was detected with COVID-19 vaccine hesitancy. Having a health insurance was explored in two studies, obtaining contrasting results [2,30]. Moreover, a high level of perceived susceptibility was assessed in one study, according to which not being aware that pregnancy increases the risk of severe illness and not being aware that pregnant women represent a priority group were more frequently reported by hesitant women [60]. Having received other vaccines was assessed in two studies, yielding contrasting results. Sutanto et al. found a significant association between having planned to receive flu vaccine or diphtheria-tetanus-pertussis vaccine and lower hesitancy. However, the other study [30] failed to find the association. Lastly, not having received a healthcare worker’s (HCWs) recommendation was more frequently reported by hesitant individuals [60]; conversely, consulting one’s own physician significantly reduced hesitancy [44].

#### 3.6.4. Pregnancy Characteristics

Gestational week was not associated with hesitancy in all the three retrieved studies [2,46,48]. Parity was assessed in four studies, obtaining contrasting results (half of them found a significant association with hesitancy [2,48], while half of them did not [38,46]). Pregnancy at risk was only assessed in one study and was not found to be associated with hesitancy [31]. Lastly, no pregnancy related issues [44] and history of reproductive issues were associated with higher rate of hesitancy [32].

#### 3.6.5. COVID-19 Related Aspects

Regarding COVID-19 related aspects, we detected the following topics: fear of COVID-19, knowledge, perceived barrier, safety and benefit of vaccination, vaccine efficacy, source of data.

Fear of COVID-19 infection was assessed in three studies, of which two found an association between no fear (or anxiety/obsession with COVID-19 symptoms) and higher hesitancy [28,50], whereas one study found an inverse association [41]. The level of knowledge on COVID-19 [31,32] and level of perceived barriers [32] did not differ among groups. High perceived vaccine benefit and trust in COVID-19 vaccine were associated with a lower rate of hesitancy [32,53]; on the contrary, distrust in vaccines was associated with a higher rate of hesitancy [28], while the perceived inefficacy of the vaccine was not associated [41]. Fear of side effects, considering both women and babies was more frequently reported by the hesitant group [60]. Lastly, the source of data was another relevant predictor factor of hesitancy: trusting rumours on social media [28] or not consulting official sources of information both increase hesitancy [41].

### 3.7. Quality Assessment

Approximately one third (n = 16) of the included studies scored equal or more than 10 and were therefore classified as high quality. Similarly, 16 studies scored equal or below 4 and were classified as low quality, while 11 studies scored between 5 and 9 and were classified as moderate quality. Quality assessment for each included study, reported item-by-item, is detailed in Supplementary Table S2. Inter-rater reliability was assessed, and discrepancy between the two reviewers was 10%. Disagreements were solved through discussion, reaching the final agreement for all the included studies.

## 4. Discussion

The systematic review of literature raises concerns about the low anti-SARS-CoV-2 vaccination coverage in pregnant and breastfeeding patients attending healthcare facilities. The more consistently cited causes of vaccine hesitancy in pregnant and breastfeeding women appear to be safety concerns and lack of causal association analysis [3]. Many included studies indicate that women are particularly concerned that vaccines might cause biological damages to either themselves [46,48,53], their child [40,41,51] or both [20,22,34,37,38]. The findings of Riad et al. [51] are suggestive, highlighting that over 60% of their sample population continued to refuse vaccination even after their physician’s recommendation and the vaccination offer in hospital setting.

These data are especially significant in consideration that these studies were carried out in hospitals or other healthcare facilities. Patients accessing healthcare would be expected to be more compliant to HCWs’ recommendations than subjects who refuse or do not actively seek medical care [62,63]. However, the included studies showed a significant level of vaccine hesitancy even in this “privileged” population. This is likely related to the multifaceted nature of vaccine hesitancy [5,64], as confirmed by several studies that have highlighted a diverse pattern of the determinants for vaccine hesitancy in this population In addition to the previously mentioned safety concerns, these reasons included religious issues [58], social disadvantage and a lack of trust in institutions [51], as well as factors related to education and occupational status [42,44,59] and misinformation about COVID-19 and its risks for pregnant women [21,27,28,39]. Older women were generally observed to have a significantly higher degree of acceptance and trust in vaccination [21,42,53,59]. Additionally, ethnicity is another factor associated with higher acceptance and lower hesitancy in the majority of included studies. However, it is important to note that the association between ethnicity and vaccine hesitancy/acceptance can depend on the specific ethnic group, cultural factors, historical experiences, and access to healthcare resources. According to our results, Afro-Caribbean, Asian ethnicity and Bedouin pregnant/breastfeeding women were more accepting, while, Asian and Sindhi pregnant/breastfeeding women were less hesitant.

These results appear to contrast with previous evidence regarding ethnicity and vaccine hesitancy/acceptance in general. These seemingly contrasting results might be due to the fact that a global vaccination campaign for COVID-19 has been launched. As a result, more language and culturally adapted communication materials have been developed by each country worldwide, as well as by international health authorities/agencies, including the World Health Organization. Moreover, free of charge access to the vaccine, and the prioritization of specific vulnerable group in terms of health condition (including pregnancy), may have facilitated the acceptancy among minorities.

From this perspective, the findings of Premji et al. [50] are particularly intriguing, as they pertain to a population of Pakistani women and also included their spouses in the data collection. According to the results of this study, the acceptance of vaccination among women in Pakistan appears to be significantly influenced by their husband’s opinion. This might be related to the religious and cultural background of these individuals. Notably, husbands of pregnant women were observed to be more supportive of COVID-19 vaccination when they had no safety concerns regarding both the woman and the child. Doubts about the efficacy of COVID-19 vaccine were also identified as a cause of vaccine hesitancy in some studies [34,48,56]. Chronic medical illness was also found to increase vaccination uptake in pregnant and breastfeeding women [45,58]. This might be attributable to a heightened awareness of morbidity and mortality associated with vaccine preventable diseases, a phenomenon observed in other populations as well [65].

Several other factors influencing vaccine hesitancy were identified, although their presence was less consistent across the included studies. Education was significantly associated with lower hesitancy levels in eight different studies, while only two identified current employment as a facilitator for vaccine acceptance. Islamic faith subjects reported higher hesitancy in one study, although the exact causes of this behaviour were not investigated. Older maternal age was investigated as a possible determinant by only ten studies, only half of which observed a consistent increase in vaccine acceptance for pregnant/breastfeeding women over 35 years of age.

In-hospital vaccination has been identified as a possible solution to low vaccination coverage [66]. Apart from the convenience of administering vaccines during a hospital stay, which eliminates the need for patients to travel long distances to access vaccination services, the idea of being constantly monitored by HCWs often serves as a facilitator of vaccine acceptance among patients, particularly in emergency areas [67,68,69]. Hospital staff also generally support this practice [70]. However, the case of COVID-19 vaccination is peculiar. The rapid development of the currently available vaccines has mitigated the growing burden of COVID-19, reducing the risk of infection, with a specific impact on symptomatic and severe disease, and has increased confidence of HCWs and the general population [71]. Despite benefits of immunization programs, hesitancy persists in specific subgroups [72,73,74] during the first phases of vaccination campaigns [75].

Our review identified an insufficient level of knowledge regarding anti-SARS-CoV-2 vaccination among pregnant women, underscoring the critical role of reliable information sources in shaping individuals’ attitude towards vaccination [43]. The widespread dissemination of misinformation concerning both COVID-19 and its vaccines could undermine vaccination coverage [76,77]. Given this complex landscape, the conventional recommendations for clearer communication with patients by HCWs [78,79] may prove inadequate to address vaccine hesitancy and promote acceptance. A structured approach, considering the level of education [55,56] is especially needed when assisting pregnant and breastfeeding women, whose acceptance of vaccination is often lower than average even for “common” vaccines [80]. It is also crucial to involve spouses and other family members in the communication process, as their opinion can be pivotal in determining the woman’s final decision regarding vaccination. This new approach to vaccination would require the use of multiple tools in a standardized manner while allowing room for personalization to accommodate various social and cultural backgrounds. The quality and quantity of information must be tailored to the patient’s ability to understand, avoiding information overload that could lead to anxiety and, ultimately, refusal [81]. Finally, when interacting with patients in a post-primary healthcare setting, emphasis should be placed on creating a safe and controlled environment where potential side effects can be handled more effectively, thereby encouraging women to accept timely vaccination.

### 4.1. Implications for Policies and Practices

The current research indicates that vaccine acceptance/hesitancy among pregnant/breastfeeding women is largely influenced by several factors, which significantly impact the final vaccine coverage rate. Therefore, our results should be taken into account to inform strategies related to public health policies and procedures. While reluctance toward vaccines primarily stems from concerns about their safety, whether considering women, the, fetus/newborn or both, many other aspects, such as educational level, source of information, and having received previous vaccination during pregnancy or at least recommendation from HCWs to receive the vaccines, positively influence acceptancy. In this perspective, simultaneously work on health education (among general public or, as in this case specific target populations) and HCWs’ training (and re-training) is of paramount importance to concurrently and correctly inform people and ensure an updated and timely education of HCWs [82]. This dual action allows, on the one hand, facilitating the public’s access to appropriate sources of information, and on the other hand, dispelling doubts among HCWs [83], especially in the case of vaccine administration among pregnant women. This is particularly important given the novelty of the vaccine and the subsequent influx of new available information [84,85,86,87].

The uncertainty among HCWs about administering vaccinations to pregnant and breastfeeding women could significantly impact their acceptance and attitudes toward vaccination. As a consequence, HCWs should equip themselves with effective information-sharing and risk communication skills [88,89]. Transparent, up-to-date, trustworthy, and timely communication is crucial for addressing concerns and misconceptions within the population [90,91]. At the same time, easy access to and understandable information about COVID-19 vaccines and vaccination might largely impact vaccine acceptance [92,93]. Therefore, taking into account our results, promoting targeted education campaigns addressing the main concerns of pregnant/breastfeeding women regarding COVID-19 safety is another aspect that healthcare systems and policymakers should consider in order to increase COVID-19 coverage and ensure the well-being of women and their child.

Given the aforementioned points, HCWs, both from the current and future generations, must stay informed about the prevailing guidelines for vaccinating pregnant women, receive training in effective communication strategies, and be actively encouraged to recommend necessary vaccines to their patients [94]. HCWs are engaged to promote immunization in different healthcare settings and to develop effective communication toolkits and educational programs about vaccination [95,96]. Moreover, fostering collaborations among HCWs, public health agencies, and citizen organizations (specialized in maternal and child health) should be encouraged and promoted to develop holistic strategies ensuring women have access to the most up-to-date evidence, policies and vaccines when recommended.

### 4.2. Strenghts and Limitations

Before we draw generalized conclusions from our findings, it is important to acknowledge certain limitations. Firstly, this is a review and, therefore, it inherited all the limitations from each primary study included. In detail, exposures and outcomes were defined and measured using different methods, increasing heterogeneity and uncertainty around the strengths of the associations. Associations were measured using different statistical methods, limiting the comparability of the results. Moreover, many studies did not report data when associations were not statistically significant, preventing the possibility to perform a statistical pooling of the results. When performing a meta-analysis, both positive and negative data should be combined in order to compute the new estimate. Furthermore, several studies did not adjust their results for potential confounders, or different confounders were considered among studies, or in some cases they didn’t explicitly delineate them. Therefore, the differences in results could be due to many methodological aspects, including sampling methods, or to real dissimilarities in study populations. Another limitation in the data is the wide variability in terms of results. This might attributable not only to methodological aspects but also the setting-country where the study was conducted. Having included subjects attending hospital or healthcare facilities might have selected those less reluctant toward vaccinations or healthcare assistance in general, thereby potentially introducing bias. Lastly, most of the included studies were cross-sectional in nature, limiting the possibility to identify a causal association. Moreover, it should be considered that studies from different countries were considered altogether. This might simultaneously represent a strength and a limitation. Actually, one hand it offers a broad overview of the state of the art, but on the other hand, it loses the specificity of each country, which is known to be peculiar in terms of vaccine hesitancy/acceptance reasons. Furthermore, we did not stratify our results based on risk of bias assessment. It implies that moderate-to-low quality studies have been compared with high quality ones. However, despite these limitations, this review boasts several merits. First, this is a systematic review conducted adhering to the PRISMA guidelines, which help in ensuring a comprehensive approach. Secondly, the protocol of the current review has been published in advance, increasing transparency. Thirdly, several outcomes were considered, offering a broad assessment of the phenomenon. Fourthly, no limits were posed about exposures, contributing to a comprehensive and exhaustive overview of predictors of vaccine acceptance/hesitancy among pregnant/breastfeeding women. Lastly, we consulted three distinct databases to capture all eligible studies, surpassing the minimum requirements set by guidelines.

## 5. Conclusions

Vaccine hesitancy in pregnant and breastfeeding women is an especially serious issue to be tackled by healthcare professionals. It is a multifaceted problem, stemming from various factors regarding women, their health and their pregnancy. While improving knowledge about vaccines in general seems fundamental to increase the public’s trust in vaccination, other interventions are required. Irrational factors such as fear of adverse events or religious concerns have been identified as important determinants of hesitancy. These issues are to be faced with personalized interventions aimed at improving communication and building a trust relationship between physicians and patient. HCWs have experienced substantial workload changes and attitude towards vaccine because of their role in managing measures to limit the spread of COVID-19.

It should also be considered that, as we have observed through our review of studies conducted in various socioeconomic and cultural settings, the reasons for hesitancy can vary significantly among different individuals. Future vaccination policies should prioritize communication on a broad range of topics to address the concerns and doubts of as many people as possible.

## Figures and Tables

**Table 1:** Main characteristics of included studies.

Author Name	Study Period	Study Design	Country	Study Settings	Recruitment Methods	Administration Method	Tool(s) Used to Assess the Outcomes	Validation (Yes/No)	Funds	Conflicts of Interests
Akhtar, 2022 [20]	October– November 2021	cross- sectional	Pakistan	Outpatient Department of Obstetrics and Gynaecology	consecutive women	n.a.	questionnaire developed ad hoc	no	n.a.	no
Aynalem, Z. B., 2022 [21]	August– September 2021	cross- sectional	Ethiopia	antenatal care at selected public health institutions	antenatal care registry	face-to-face	questionnaire developed ad hoc	yes, Cronbach’s alpha = 0.87	no	no
Bagalb, 2022 [22]	November 2021– February 2022	cross- sectional	Saudi Arabia	maternity department of the tertiary care setting	snow ball technique	self- administered	questionnaire developed ad hoc	yes, pre-tested	n.a.	no
Blakeway, 2022 [23]	March 2020–July 2021	cohort	United Kingdom	University Hospitals (London)	n.a.	n.a.	electronic medical records	n.a.	no	no
Carbone, 2021 [24]	January 2021	cross- sectional	Italy	Two University teaching hospitals (Naples and Rome)	consecutive women	on-line	questionnaire developed ad hoc	no	n.a.	n.a.
Chawanpaiboon, 2023 [25]	January–April 2022	cohort	Thailand	postpartum ward	consecutive women	n.a.	questionnaire developed ad hoc	yes, no further details	n.a.	no
Chekol Abebe, E., 2022 [26]	March 2022	cross- sectional	Ethiopia	Debre Tabor public health institutions	consecutive women	face-to-face	questionnaire developed ad hoc	no, developed based on literature	n.a.	no
Citu, C. 2022 [27]	January–May 2022	cross-sectional	Romania	Obstetrics and Gynecology Clinic	convenience sampling	on-line	questionnaire developed ad hoc	no	no	no
Citu, I. M., 2022 [28]	October–December 2021	cross-sectional	Romania	Obstetrics and Gynecology Clinic of the Timisoara Municipal Emergency Hospital	convenience sampling	on-line	VAX (Vaccination Attitude Examination) scale	yes, no further details	no	no
Davies, 2022 [29]	October–November 2021	cross-sectional	England	Hospital maternity department (antenatal clinics, maternity triage and maternity day unit)	consecutive women	on-line	questionnaire developed ad hoc	no	no	no
DesJardin, M., 2022 [30]	September–October 2021	cross-sectional	USA	Prenatal care at a central New York regional Maternal–Fetal Medicine clinic	consecutive women	face-to-face	questionnaire developed ad hoc	no	no	no
Ercan, A., 2022 [31]	March–April 2021	cross-sectional	Turkey	Outpatient Obstetrics Clinics of İstanbul Training and Research Hospital	n.a.	n.a.	questionnaire developed ad hoc	yes, Cronbach’s alpha 0.82	no	no
Firouzbakht, M., 2022 [32]	October 2021–January 2022	cross-sectional	Iran	public healthcare centers in the north of Iran	convenience sampling	self-administered	questionnaire developed ad hoc	no	yes	no
Geoghegan, S., 2021 [33]	December 2020–January 2021	cross-sectional	Ireland	prenatal care in hospital- based public, private, and semi-private clinics, and in community-based midwife-lead clinics	consecutive women	on-line	questionnaire developed ad hoc	yes, pre-tested	yes	no
Getachew, T., 2022 [34]	June 2021	cross-sectional	Ethiopia	public hospitals of Dire Dawa city	random sampling techniques	face-to-face	questionnaire developed ad hoc	yes, pre-tested	no	no
Goncu Ayhan, S., 2021 [35]	January–February 2021	cohort	Turkey	Ankara City Hospital	consecutive women	face-to-face	n.a.	no	n.a.	no
Gupta, A., 2022 [36]	July–August 2021	cross-sectional	India	Gynecology and Obstetrics Department of a tertiary care institute	antenatal care registry	phone calls	questionnaire developed ad hoc	n.a.	no	no
Husain, 2022 [37]	September 2021–February 2022	cross-sectional	England	antenatal clinic (general hospitals)	consecutive women	self-administered	questionnaire developed ad hoc	yes, pre-tested	no	n.a.
Karagöz, 2022 [38]	January–April 2022	cross-sectional	Turkey	local hospital (Samsun Training and Research Hospital Gynecology and Obstetrics Outpatient Clinics)	consecutive women	face-to-face	questionnaire developed ad hoc	yes, pre-tested	no	no
Kiefer, 2022 [2]	March–April 2021	cross-sectional	USA	general obstetrics, midwifery and maternal–fetal medicine clinics	consecutive women	face-to-face	the Attitude toward COVID-19 vaccine scale	yes, no further details	n.a.	no
Kumari, 2022 [18]	February–April 2022	cross-sectional	India	antenatal clinic	consecutive women	face-to-face	questionnaire developed ad hoc	n.a.	n.a.	no
Miraglia Del Giudice, 2022 [41]	September 2021–May 2022	cross-sectional	Italy	two public hospitals	random sampling techniques	face-to-face	questionnaire developed ad hoc	yes, by opinion from experts	n.a.	no
Mose, 2021 [42]	February–March 2021	cross-sectional	Ethiopia	hospital	consecutive women	face-to-face	questionnaire developed ad hoc	yes, pre-tested	no	no
Mose, A. and A. Yeshaneh 2021 [43]	January 2021	cross-sectional	Ethiopia	Antenatal Care Clinic hospital	random sampling techniques	face-to-face	n.a.	yes, Cronbach’s alpha (α) = 0.79	no	no
Mustafa, Z. U., 2022 [44]	December 2021–January 2022	cohort	Pakistan	antenatal clinics from 7 hospitals	consecutive women	face-to-face and on-line	questionnaire developed ad hoc	no, developed based on literature	no	no
Nazzal, 2022 [45]	October–November 2021	cross-sectional	Palestine	health care facilities	n.a.	face-to-face	questionnaire developed ad hoc	yes, pre-tested	no	no
Nemat, A., 2022 [46]	July–August 2021	cross-sectional	Afghanistan	gynecology wards of several hospitals in Kabul	consecutive women	face-to-face	questionnaire developed ad hoc	yes, Cronbach’s alpha coefficients = 0.74	n.a.	n.a.
Nguyen. 2021 [47]	January–February 2021	cross-sectional	Vietnam	hospital (central and provincial)	consecutive women	face-to-face	questionnaire developed ad hoc	n.a.	n.a.	n.a.
Odabas, 2022 [39]	September 2021–January 2022	cross-sectional	Turkey	public hospital	consecutive women	face-to-face	questionnaire developed ad hoc	no	no	no
Oluklu, D., 2021 [48]	February–March 2021	cross-sectional	Turkey	Ankara City Hospital	n.a.	face-to-face	questionnaire developed ad hoc	no	n.a.	no
Pairat, 2022 [49]	July–September 2021	cohort	Thailand	Antenatal care	consecutive women	self-administered	questionnaire developed ad hoc	no	yes	no
Premji, 2022 [50]	July–September 2020	cross-sectional	Pakistan	4 centres of Aga Khan Hospital for Women and Children	within the ongoing prospective longitudinal Pakistani cohort study	phone calls	questionnaire developed ad hoc	no	yes	no
Riad, A., 2021 [51]	August–October 2021	cross-sectional	Czechia	Gynecologic clinic of the University Hospital Brno	consecutive women	self-administered	questionnaire developed ad hoc	yes, pre-tested	yes	no
Siegel, 2022 [52]	June–August 2021	cross-sectional	USA	health centers	consecutive women	n.a.	questionnaire developed ad hoc	no	n.a.	n.a.
Sutanto, 2022 [53]	August–September 2021	cross-sectional	USA	hospital south Texas	consecutive women	n.a.	questionnaire developed ad hoc	no, developed based on literature and considering the Health Believe Model	no	no
Sznajder, K. K., 2022 [54]	May–December 2020	cross-sectional	USA	Mid-size academic medical center in Central Pennsylvania	consecutive women	on-line	questionnaire developed ad hoc	n.a.	yes	no
Tao, 2021 [55]	November 2020	cross-sectional	China	obstetric clinics of 6 hospitals	multistage sampling approach	n.a.	questionnaire developed ad hoc	yes, Cronbach’s α coefficient= 0,81	yes	no
Tatarevic, T., 2022 [56]	May–October 2021	cross-sectional	Croatia	antenatal clinic in two teaching hospitals	consecutive women	face-to-face	questionnaire developed ad hoc	no	no	no
Taye, E. B., 2022 [57]	August–September 2021	cross-sectional	Ethiopia	Antenatal and postnatal cares in Central Gondar Zone public hospitals	random sampling techniques	face-to-face	questionnaire developed ad hoc	yes, pre-tested	no	no
Tefera, 2022 [58]	January 2022	cross-sectional	Ethiopia	public hospitals	multistage sampling approach	face-to-face	questionnaire developed ad hoc	yes, pre-tested	n.a.	no
Wainstock, T., 2023 [59]	January–September 2021	cohort	Israel	Soroka University Medical Center	antenatal care registry	n.a.	electronic medical records	yes	n.a.	no
Ward, 2022 [60]	September–October 2021	cross-sectional	Australia	maternity units	consecutive women	on-line	questionnaire developed ad hoc	no	n.a.	no
Yoon, H., 2022 [61]	January–April 2022	cross-sectional	South Corea	Mix of public and private clinics or hospitals	consecutive women	face-to-face and on-line	questionnaire developed ad hoc	yes, pre-tested	yes	no

n.a: not available.

**Table 2:** Main characteristics of studied population.

Author Name	Main Characteristics of the Population	Women’s Age (Mean ± SD, or Range or %)	Sample Size	Attrition (Not Competition Rate)	Adjustment
Akhtar, 2022 [20]	Pregnant and breastfeeding women	27.15 ± 4.788 years	500 (249 pregnant, 251 breast feeding)	28%	no
Aynalem, Z. B., 2022 [21]	Pregnant women	30.7 ± 5.86 years	525	2.9%	yes but not specified
Bagalb, 2022 [22]	Pregnant and breastfeeding women	n.a.	300 (53.3% pregnant and 46.7% breastfeeding/lactating mothers)	20%	no
Blakeway, 2022 [23]	Pregnant women	30–37 years	1328	26.8%	yes but not specified
Carbone, 2021 [24]	Pregnant and early postpartum patient	34 (range 31−37.25) years	142 (83.8% pregnant and 16.2% early postpartum period)	15.5%	not applicable, chi-squared test
Chawanpaiboon, 2023 [25]	Breastfeeding women	30.9 (range 15–43) years	400	n.a.	yes but not specified
Chekol Abebe, E., 2022 [26]	Pregnant women	32.3 ± 4.14 (range 18–50) years	634	0%	yes but not specified
Citu, C. 2022 [27]	Pregnant women	n.a.	345	16.3%	no
Citu, I. M., 2022 [28]	Pregnant women	30.6 ± 7.2 years	184	n.a.	knowledge, history of medical diseases, and history of reproductive problems
Davies, 2022 [29]	Pregnant women	n.a.	202	n.a.	not applicable, chi-squared test
DesJardin, M., 2022 [30]	High-risk pregnant women	n.a.	157	22%	hierarchical Bayesian model
Ercan, A., 2022 [31]	Pregnant women	18–49 years	250	n.a.	knowledge, history of medical diseases, and history of reproductive problems
Firouzbakht, M., 2022 [32]	Pregnant women	20–35 years	352	8%	knowledge, history of medical diseases, and history of reproductive problems
Geoghegan [33]	Pregnant women	18–45 years	300	12.3%	no
Getachew, T., 2022 [34]	Pregnant women	Mean age 28.92 ± 6.7 years	645	n.a.	yes but not specified
Goncu Ayhan, S., 2021 [35]	Pregnant women	27.99 ± 5.6	300	n.a.	not applicable, correlation analysis
Gupta, A., 2022 [36]	Pregnant not fully vaccinated before pregnancy	28.3 ± 5.5 ye	163	43%	yes but not specified
Husain, 2022 [37]	Pregnant women	32.0 (17–44)	441	n.a.	not applicable, chi-squared test
Karagöz, 2022 [38]	Pregnant women	28.7 ± 5.3 years	247	11.7%	not applicable, chi-squared test
Kaya Odabas, 2022 [39]	Pregnant and postpartum individuals	29 years (SD: 5.38 years)	456	5.9%	age, parity, race, trimester of pregnancy, and chronic comorbidities
Kiefer, 2022 [2]	Pregnant women	21–30 years = 79.69%	298	n.a.	yes but not specified
Kumari, 2022 [18]	Pregnant and breastfeeding	32.2 ± 5.4 (range 19–46) years	385	5.2%	yes but not specified
Miraglia Del Giudice, 2022 [41]	Lactating mothers	25 ± 0.42 years	630	n.a.	yes but not specified
Mose, 2021 [42]	Pregnant women	25.38 ± 3.809 years	396	0	yes but not specified
Mose, A. and A. Yeshaneh 2021 [43]	Pregnant women	29.1 years	405	37.7%	no
Mustafa, Z. U., 2022 [44]	Pregnant women	n.a.	860	9.5%	yes but not specified
Nazzal, 2022 [45]	Pregnant women	27.24 ± 5.698 years	491	4.3%	not applicable, chi-squared test
Nemat, A., 2022 [46]	Pregnant women	29.4 ± 5.0 years	651	3.6%	no
Nguyen. 2021 [47]	Pregnant women	26.33 ± 4.96 years	400	n.a.	no
Oluklu, D., 2021 [48]	Postpartum women	28.69 ± 5.4 years	412 (88.1% breastfeeding)	n.a.	not applicable, spearman correlation
Pairat, 2022 [49]	Pregnant women	28 years (IQR 23–33 years)	171	2.8%	no
Premji, 2022 [50]	Postpartum women	26–30 years	941	4.9%	no
Riad, A., 2021 [51]	Pregnant and lactating	31.48 ± 4.56 (range 19–44) years	362 (278 pregnant and 84 lactating)	9.7%	yes but not specified
Siegel, 2022 [52]	Pregnant and postpartum	vaccinated 33.0 ± 4.5; unvaccinated 31.4 ± 5.6)	473	0.8%	no
Sutanto, 2022 [53]	Pregnant women	31 years among vaccinated, 28 years among not vaccinated	109	8.4%	no
Sznajder, K. K., 2022 [54]	Pregnant women	<35 years 80%>35 years 20%	196	5.7%	yes but not specified
Tao, 2021 [55]	Pregnant women	55.4% equal or below 30 years old	1392	n.a.	age group, region, education, occupation, monthly household income per capita), health status (gravidity, parity, gestational trimester, history of adverse pregnancy outcomes, history of chronic disease, history of influenza vaccination, and gestational complications), total knowledge score on COVID-19 (as continuous variable), health belief (susceptibility, severity, barriers, benefits, and cues to action)
Tatarevic, T., 2022 [56]	Pregnant women	31 (IQR = 27–36) years	430	9%	not applicable, chi-squared test
Taye, E. B., 2022 [57]	Pregnant and postnatal women	18–25; n = 19526–35; n = 29036–48, n = 34	519 (360 pregnant and 159 postnatal)	1.5%	yes but not specified
Tefera, 2022 [58]	Pregnant women attending antenatal care	<20 up to 49 years	702	0%	yes but not specified
Wainstock, T., 2023 [59]	Pregnant (women who delivered during the study period)	20–35 years	7017	n.a.	yes but not specified
Ward, 2022 [60]	Pregnant women	31.9 years	218	n.a.	not applicable, chi-squared test
Yoon, H., 2022 [61]	Pregnant or postpartum women	Among acceptant 33.28 ± 4.70 years; among refusal 33.65 ± 3.77 years	533 (87.8% pregnant and 12.2% postpartum)	15.4%	maternal age, occupation, and pregnancy period

IQR: Interquartile range; n.a.: not available; SD: standard deviation.

**Table 3:** Predictors of vaccine acceptance and vaccine hesitancy.

	Predicators of Vaccine Acceptance		Predictors of Vaccine Hesitancy	
	Significant	Not Significant	Significant	Not Significant
Action	High level cues to action * aOR: 15.70 (8.28–29.80) [55]		Cues to action aOR: 0.621 (0.516–0.574) [32]	Self-efficacy [32]
Age	Younger age aOR: 1.87 (1.20–2.93) [55]; 34–41 y aOR: 1.46 (1.22–5.13) [43]; age (continuous scale) aOR: 1.03 (1.02–1.05) [59]; age ≥ 35 y aOR: 5.68 (1.78–18.17) [21]; 30–35 y OR: 2.43 (1.25–4.75) [33]	Maternal age [23,26,35,42,45]	Age > 25 y aOR: 0.30 (0.17–0.54) [2]; age gravidity significantly different among groups [48]	Age [30,31,38]
Alcohol/Drugs		Alcohol [23]		Use of drugs [30],
Attitude	positive attitude aOR: 1.59 (1.09, 2.31) [58]; positive attitude aOR: 8.54 (5.18–14.08) [57]; good attitude aOR = 2.128, (1.348–3.360) [21], positive attitude significantly different among groups [24],	Attitude [42,43]		
Barrier	low level of perceived barriers aOR: 4.76 (2.23–10.18) [55]			Perceived barriers [32],
Benefit	high level of perceived benefit aOR: 2.18 (1.36–3.49) [55]; perceived benefits aOR: 1.1 (1.06–1.16) [45]; risk/benefit ration 15.52 (2.78–86.80) [51]		Perceived benefits aOR: 0.700 (0.594–0.825) [32]; believe that vaccine will protect against COVID-19 OR: 0.1 (0.04–0.28) [53]; confidence in COVID-19 vaccine OR: 0.04 (0.02–0.13) [53]; feel confident in making a decision OR: 0.23 (0.07–0.73) [53]; not believing in vaccines aOR: 3.15 (2.80–3.49) [28]; vaccination not needed OR = 2.54 (1.11–5.75) [22]	
BMI		BMI [23]		
COVID-19 Fear	Worry about COVID-19 infection OR: 1.55 (0.55, 4.40) [49]; Fearing the severity of COVID-19 disease OR: 0.68 (0.34–0.82) [27]; fear of COVID-19 disease aOR: 3.46 (2.16–5.52) [57]	Fear of COVID-19 infection [41,61]	Not believing in the existence of the SARS-CoV-2 virus aOR: 2.67 (2.12–3.04) [28], no fear aOR = 1.89 (1.54–2.27) [28], lower fear of COVID-19 infection OR: 0.77 (0.64–0.93) [41], no COVID-19 anxiety symptoms OR: 2.32 (1.26–4.28) [50]; no obsession with COVID-19 symptoms OR: 2.22 (1.30–3.77) [50]	Perceived threat [32],
Data Availability	unavailability of data regarding safety during pregnancy and breast-feeding [20]; no need to receive information on COVID-19 vaccine 0.41 (0.21–0.79) [41]; feel the vaccine was rushed OR: 0,16 (0.10–0.27) [52]; believe people of their race were included in trials OR: 2.65 (1.79–3.92) [52]			
Education	lower level of education (aOR: 2.49, (1.13–5.51) [55]; higher education OR: 0.81 (0.62–0.95) [27]; higher educational level 1.92 (1.03–3.57) [41]; higher educational level aOR: 4.2 (2.1–8.5) [34]; higher educational level aOR 3.48 (1.52–7.95) [43]; higher educational level 2.8 (1.51–4.21) [42]; higher educational level 5.99 (1.12–32.16) [51]; level of education significantly differed between groups [56]	Educational status [20,21,36,45,54]	Higher educational level aOR: 0.05 (0.02–0.13) [2]; lower educational level OR: 0.38 (0.15–0.92) [41]; lower education level OR: 3.42 (1.24–9.45) [22]; lower educational level aOR: 4.93 (2.47–9.83) [25]	Educational level [30,31,38,46,50]
Efficacy	Confidence in vaccine efficacy OR = 1.85 (0.38, 9.11) [49]; believe vaccine will protect them against COVID-19 OR: 0 10.75 (6.73–17.17) [52]; believe vaccine will protect their baby from COVID-19 OR: 6.36 (4.16–9.73) [52]		Believe that vaccine during pregnancy increase the newborn’s immunity aOR: 0.28 (0.08–0.98) [25]	Believe that vaccine is ineffective [41]
Ethnicity	Afro-Caribbean 0.27 (0.06–0.85) [23]; Asian ethnicity significantly more frequently reported among vaccinated women [29]; Bedouin aOR 0.20 (0.18–0.23) [59]		Asian aOR: 0.11 (0.02–0.57) [2]; Sindhi OR: 0.43 (0.20–0.93) [50]	Ethnicity [30]
Facility	Availability of vaccination centres nearby OR: 0.87 (0.63–0.99) [27]			
Government Trust	awareness that COVID-19 vaccine has been approved by the government aOR: 3.03, CI: 1.45–6.36) [40]; Trusting the government OR: 0.83 (0.59–0.99) [27]; trust vaccine features OR: 6.52 (4.30–9.91) [52]			
Health	chronic medical illness aOR: 2.41 (1.28, 4.54) [58]; underlying medical condition aOR: 2.1; (1.1–4.1) [45], 2022; diabetes 10.5 (1.74–8.32) [23]; history of chronic diseases 2.52 (1.34–4.7) [34]; having a pre-existing chronic disease aOR: 3.131 (1.700–5.766) [21],	Health status [41]; health condition [43]; comorbidities [35,36,59]; obesity [59]; diabetes [59]		Chronic comorbidities [2]; disease history [32]
Husband	having a husband who favoured COVID-19 vaccination OR: 4.82 (2.34, 9.94) [49]; living with husband and children OR: 0.5 (0.28; 0.9) [47]; husbands’ educational level aOR: 1.99 (1.09, 3.64) [58]	Marital status [21]		Marital status [30], husband’s educational level [46]
Infection	history of COVID-19 infection OR: 4.33 (2.31–8.12) [41]	History of COVID-19 infection [34,36,45]; antenatal COVID-19 [23]; tested COVID-19 positive [21]	History of COVID-19 infection aOR: 0.47 (0.24–0.90) [25]	History of COVID-19 [30]; tested COVID-19 positive [50]
Insurance	private health insurance OR: 0.46 (0.26; 0.82) [47]		Public health insurance aOR: 3.93 (2.41–6.43) [2]; insurance type correlated [30]	
Knowledge	high knowledge score on COVID-19 aOR: 1.05, (1.01–1.10) [55]; Knowledge on COVID-19 vaccine aOR: 2.0; (1.2–3.1) [45]; good knowledge aOR 5.95 (3.15–7.07) [43]; good knowledge about vaccine aOR: 2.6 (1.84–3.47) [42]; good COVID-19 vaccine knowledge aOR: 9.56 (62.31, 39.53) [36]; good knowledge about COVID-19 vaccine aOR = 2.391, (1.144, 4.998) [21];	Knowledge on COVID-19 infection [45]		COVID-19 knowledge [31,32]
Employment	employment aOR: 5; (3.1–8.1) [45]; employed 2.22 (1.02–4.81) [54]; feeling overloaded 2.18 (1.02–4.68) [54]	Employment [20,21,34,43,51,61]; work related stress [54]	Employment OR: 4.47 (2.31–8.64) [44]	Employment [30,31,38]
Pregnancy	gravida > 2 aOR: 1.84 (1.30–2.61) [40]; late pregnancy (aOR: 1.49, (1.03–2.16) [55], recurrent pregnancy loss aOR: 0.78 (0.61–0.99) [59]; pregnancy status statistically significant different among groups [24], insufficient prenatal care aOR: 0.36 (0.30–0.42) [59]; infertility treatment aOR: 1.47 (1.18–1.83) [59]; poor obstetric history aOR: 0.65 (0.49–0.87) [59]; parity statistically significant different among groups [24]	Gravity [35]; number of antenatal care visit [21]; pregnancy a risk [41]; number of pregnancy [43]; history of abortion [21], parity [26]; previous pregnancy [51]; multiple gestation [59]; number of pregnancy [56]	Multiparity aOR: 2.07 (1.24–3.46) [2]; parity significantly different among groups [48]; childbirth during pandemic OR: 2.16 (1.17–4.00) [50]; no pregnancy-related issues OR: 6.02 (2.36–15.33) [44]; history of reproductive problems aOR: 2.327; (1.262 to 4.292) [32]	number of pregnancy [38], parity [46]; high risk pregnancy [31]
Gestational Week	Third trimester of pregnancy OR: 0.54 (0.28–0.86) [27]; later gestational age (OR 3.74, 95% CI 1.64–8.53) [33]; gestational week significantly differed among groups [56]; second trimester of pregnancy aOR: 7.35 (1.54–35.15) [61]; gestational week (third trimester): aOR 6.50 (1.21–35.03) [51]	Gestational week [35,45]		Gestational week [2,48] gravidity [46]
Prevention	good practice of COVID-19 preventive measures aOR: 1.59 (1.09, 2.31) [58]; good practice aOR: 9.15 (8.73–12.19) [43]; good adherence to COVID-19 mitigation measures 3.2 (1.91–5.63) [42]			
Residency	Western region aOR: 2.73, (1.72–4.32), [55]; urban area of residence OR: 0.86 (0.59–0.98) [27]; resident in urban area aOR: 2.03 (1.09–3.77) [57]; urban residency aOR: 2.5 (1.62–3.91) [42]	Living in rural area [34]		Resident area [46]
Religion	Muslim religion aOR = 0.27 (0.12–0.61) [40]	Religion [20]		
Safety	vaccine being harmful during pregnancy and breast-feeding for mother & baby [20]; confidence in vaccine safety OR: 1.66 (0.35, 7.97) [49]; fear of side effect aOR: 0.09 (0.02–4.98) [36]; COVID-19 vaccine to pregnant women would benefit her baby aOR: 18.47 (2.76–123.52) [36]; considering COVID-19 vaccine safe for both mother and fetus significantly different among groups [26], fear of side effect for pregnant OR: 0.18 (0.12–0.27) [52]; fear of side effect for baby OR: 0.17 (0.11–0.25) [52]; believe the vaccine will cause them COVID-19 infection OR: 0.21 (0.08–0.56) [52]; worried about toxins in the vaccine OR: 0.22 (0.13–0.38) [52]	Awareness that vaccine could protect fetus [61]	Fear of side effects for mother and newborn were significantly more frequently reported by unvaccinated women [60]; fear of side effects OR: 2.92 (1.09–7.79) [22]	
Smoking		Smoking [23,59],	Tobacco use aOR: 3.20 (1.46–7.01) [2]	Smoking [31]
Cohabitation	Seeing more people getting vaccinated OR: 0.75 (0.33–0.88) [27]; living with a vaccinated family member significantly more frequently reported among vaccinated women [29]; living with a vaccinated member aOR: 2.43 (1.06–5.59) [61]; positive correlation between acceptance and number of school-age children [35]; having contact history with COVID-19 diagnosed people aOR: 7.724 (2.183, 27.329) [21]	Having a family member/friend lost to COVID-19 [21]; number of householders [35]; householders > 65 y [35]	Number of households significantly different among groups [48]; number of school children significantly different among groups [48]; know other pregnant women vaccinated OR: 0.26 (0.09–0.76) [53]; considering vaccination only if many people are vaccinated OR: 0.39 (0.19–0.81) [22]; need to consult relative before receiving the vaccine aOR: 2.58 (1.30–5.09) [25]	Number of housholds with comorbidities [48]
Income	lower income aOR: 0.10 (0.02–0.40) [23]	Socioeconomic status [20]; income [34,35,59]	Low income aOR: 2.06 (1.74–2.71) [28]	Income [46]; living situation [30]; economic status [31]
Susceptibility	high level of perceived susceptibility aOR: 2.18 (1.36–3.49) [55]		Not being aware that pregnant women are a priority group more frequently reported by unvaccinated women [60]; no awareness that pregnancy increased the risk of severe illness more frequently reported by unvaccinated women [60]	
Travelling	Caring about travelling OR 0.76 (0.40–0.87) [27]			
Source of Data	Official source of information OR: 2.92 (1.58–5.42) [41]; being exposed to COVID-19 vaccine information aOR: 2.2 (1.41–3.57) [34];		Trusting rumours on social media aOR: 2.38 (1.90–2.94) [28]; not official source of information OR: 6.18 (2.53–15.09) [41]; social media news on vaccine safety aOR: 0.32 (0.13–0.84) [25]	
Hcws’ Recommendation for Vaccination	having received recommendation from HCWs more frequently reported among vaccinated women [29]; immunization counselling received aOR: 3.4 (1.95–5.91) [42]; received vaccine recommendation from HCWs aOR: 3.41 (2.05–5.65) [61]; having received information form HCWs aOR: 4.36 (1.28–14.85) [51]		Having not received recommendation by HCWs more frequently reported by unvaccinated women [60]; consulted their doctors OR: 0.12 (0.04–0.35) [44]; recommendation from physician 0.34 (0.15–0.77) [22]	
Having Received/Planned Other Vaccinations	having received influenza vaccine aOR 4.82 (2.17–10.72) [54]; willingness to receive pertussis and influenza vaccine were significantly different among groups [26]; received influenza or pertussis vaccine during pregnancy statistically significant different among groups [24]		Planning to receive flu vaccine during pregnancy OR: 0.11 (0.04–0.33) [53], planning to receive Tdap during pregnancy OR: 0.29 (0.1–0.87) [53],	Other vaccine [30]

* defined as events, people, or things that trigger people to change behavior. HCWs: healthcare workers.

## Data Availability

All data are reported in the current manuscript.

## Supplementary Materials

The following supporting information can be downloaded at: https://www.mdpi.com/article/10.3390/vaccines11111697/s1, Table S1. Main reasons for vaccine hesitancy; Table S2. Item-by-item quality assessment for each included study, reported in alphabetical order. Using the Joanna Briggs Institute quality assessment tool.
