# Supplementary material for: COVID-19 Vaccine Knowledge, Attitude, Acceptance and Hesitancy among Pregnancy and Breastfeeding: Systematic Review of Hospital-Based Studies
Source: Vaccines (Basel). 2023 Nov 7;11(11):1697. doi: 10.3390/vaccines11111697 (PMC10675759; doi:10.3390/vaccines11111697)
Supplement: Supplementary file 1 [file vaccines-11-01697-s001.zip › vaccines-2642222-supplementary.pdf]

Supplementary Table S1. Main reasons for vaccine hesitancy with reported percentage. Articles are reported in alphabetical order.

**Reference**	**Vaccine safety**	**Vaccine efficacy**	**Lack of data**	**Rumours on social media**	**HCW' s recommendation not received**	**Family do not allow**	**Previous COVID-19 infection**
Chekol	61.3% for baby; 59.1% for women	58.0%					
Citu I				78.1%			
DesJardin	60%		66%				
Ercan	67.8% for baby, 59.6% for women		52.5%				
Goncu	41.7% for baby		65.6%				
Husain	80.3% for baby; 44.4% for women		60.6%				
Karagöz	50.0%				14.9%		
Mustafa	7.9%*		6.7%			10.6%	
Nemat	73.4% for baby; 20.5% for women°						39.3%
Odabas	22.5% for baby, 31.4% for women		20.4%				
Oluklu		32.4%	76%				
Tao	47.0%	44.1%					
Tatarević	75% for baby; 73% for women	41%					
Yoon	91.7% for baby	42.6%					
*death; ° infertility							

Supplementary Table S2. Item-by-item quality assessment for each included study, reported in alphabetical order. Using the Joanna Briggs Institute quality assessment tool.

	Item 1	Item 2	Item 3	Item 4	Item 5	Item 6	Item 7	Item 8	Overall score	Overall appraisal
Akhtar, 2022	2	2	-2	2	-2	-2	-2	2	0	low
Aynalem,2022	2	2	-2	2	2	2	2	2	12	high
Bagalb, 2022	2	2	-2	2	-2	-2	2	2	4	low
Blakeway, 2022	2	2	2	2	2	2	-1	2	13	high
Carbone, 2021	2	2	-2	2	0	0	-2	2	4	low
Chawanpaiboon, 2023	2	2	-2	2	2	2	2	2	12	high
Chekol Abebe, 2022	2	2	-2	2	2	2	-2	2	8	moderate
Citu, C. 2022	2	2	-2	2	-2	-2	-2	2	0	low
Citu, I. M., 2022	2	2	-2	2	2	2	2	2	12	high
Davies, 2022	2	2	-2	2	0	0	-2	2	4	low
DesJardin, 2022	2	2	-2	2	2	2	-2	2	8	moderate
Ercan, 2022	2	2	-2	2	2	2	2	2	12	high
Firouzbakht, 2022	2	2	-2	2	2	2	-2	2	8	moderate
Geoghegan, 2021	2	2	-2	2	-2	-2	2	2	4	low
Getachew, 2022	2	2	-2	2	2	2	2	2	12	high
Goncu Ayhan, 2021	2	2	-1	2	0	0	-2	2	5	moderate
Gupta, 2022	2	2	-2	2	2	2	-1	2	9	moderate
Husain, 2022	2	2	-2	2	0	0	2	2	8	moderate
Karagoz, 2022	2	2	-2	2	0	0	2	2	8	moderate
Kaya Odabas, 2022	2	2	-2	2	-2	-2	2	2	4	low
Kiefer, 2022	2	2	-2	2	2	2	-1	2	9	moderate
Kumari, 2022	2	2	-2	2	2	2	2	2	12	high
Miraglia Del Giudice, 2022	2	2	-2	2	2	2	2	2	12	high
Mose, 2021	2	2	-1	2	2	2	2	2	13	high
Mose, and Yeshaneh 2021	2	2	-2	2	2	2	-2	2	8	moderate
Mustafa, 2022	2	2	-2	2	-2	-2	2	2	4	low
Nazzal, 2022	2	2	-2	2	2	2	2	2	12	high
Nemat, 2022	2	2	-2	2	0	0	-1	2	5	moderate
Nguyen, 2021	2	2	-2	2	-2	-2	-2	2	0	low
Oluklu, 2021	2	2	-2	2	0	0	-2	2	4	low
Pairat, 2022	2	2	-2	2	-2	-2	-2	2	0	low
Premji, 2022	2	2	-2	2	-2	-2	-2	2	0	low

Riad, 2021	2	2	-2	2	2	2	2	2	12	high
Siegel, 2022	2	2	-2	2	-2	-2	-2	2	0	low
Sutanto, 2022	2	2	-2	2	-2	-2	-2	2	0	low
Sznajder, 2022	2	2	-2	2	2	2	-1	2	9	moderate
Tao, 2021	2	2	-2	2	2	2	2	2	12	high
Tatarevic, 2022	2	2	-2	2	0	0	-2	2	4	low
Taye, 2022	2	2	-2	2	2	2	2	2	12	high
Tefera, 2022	2	2	-2	2	2	2	2	2	12	high
Wainstock, 2023	2	2	2	2	2	2	2	2	16	high
Ward, 2022	2	2	-2	2	0	0	-2	2	4	low
Yoon, 2022	2	2	-2	2	2	2	2	2	12	high
